# Monthly Record of Deaths in the City of Buffalo

**Published:** 1854-07

**Authors:** Jas. M. Newman

**Affiliations:** Health Physician


ART. XII.—*Record of Mortality in the City of Buffalo, for the Month of May*, 1854. By J. M. NEWMAN, Health Physician.

| DISEASES. | No. | Males. | Females. |
|---|---|---|---|
| Accidens, | 2 | 2 | |
| Apoplexia, | 2 | 1 | 1 |
| Angina Gangraenora, | 2 | 1 | 1 |
| Asphyxia Immersorum, | 8 | 5 | 1 |
| Bronchitis, | 1 | 1 | |
| Cholera Infantum, | 1 | 1 | |
| " Morbus, | 1 | 1 | |
| " Asphyxia, | 1 | | 1 |
| Cynanche Trachealis, | 7 | 3 | 4 |
| Debilitas, | 6 | 5 | 1 |
| Degeneratio Hepatitis, | 1 | 1 | |
| Dysenteria, | 1 | | 1 |
| Eclampsia, | 10 | 3 | 7 |
| Enteritis, | 1 | | 1 |
| Febris Biliosa, | 2 | 2 | |
| " Scarlatina, | 4 | 1 | 3 |
| " Typhoid, | 3 | 1 | 2 |
| " Typhus, | 1 | 1 | |
| Hæmorrhagia Intestinorum, | 1 | 1 | |
| Hydrops, | 2 | 2 | |

### REGISTER OF MORTALITY—(Continued.)

| DISEASES. | No. | Males. | Females. |
|---|---|---|---|
| " Cerebri, | 8 | 4 | 3 |
| Hyperæmia Cerebralis, | 2 | 1 | 1 |
| " Pulmonalis, | 2 | | 2 |
| Laryngitis, | 1 | 1 | |
| Marasmus, | 6 | 3 | 3 |
| Morbus Cordis, | 4 | 4 | |
| Natus Mortuus, | 6 | | |
| Negligentia, | 3 | 2 | |
| Pneumonia, | 2 | 1 | |
| Rubeola, | 1 | | 1 |
| Paralysis, | 1 | | 1 |
| Phlebitis, | 30 | 14 | 16 |
| Pleurisy, | 1 | | 1 |
| Phthisis Pulmonalis, | 9 | 9 | |
| Phrenitis. | 8 | 2 | 6 |
| Rheumatism, | 1 | | 1 |
| Scrophula, | 2 | 1 | 1 |
| Senectus, | 3 | 2 | 1 |
| Tussis Catarshalis, | 1 | 1 | |
| Varicella, | 1 | 1 | |
| Variola, | 1 | | 1 |
| Ignotus, | 27 | 16 | 7 |
| | 177 | | |

| | |
|---|---|
| Males, | 94 |
| Females, | 68 |
| Sex not given, | 15 |
| Total, | 177 |

### AGES.

| | | | |
|---|---|---|---|
| Still-born, | 6 | Between 12 years and 20 years, | 12 |
| 1 day, | 1 | " 20 " " 30 " | 11 |
| Between 1 day and 15 days, | 10 | " 30 " " 40 " | 15 |
| " 15 days and 30 days, | 4 | " 40 " " 50 " | 6 |
| " 1 month and 3 months, | 8 | " 50 " " 60 " | 7 |
| " 3 months and 6 months, | 12 | " 60 " " 70 " | 6 |
| " 6 " " 9 " | 2 | " 70 " " 80 " | 1 |
| " 9 " " 12 " | 6 | " 80 " " 90 " | 1 |
| " 1 year and 3 years, | 32 | " 90 " "100 " | 1 |
| " 3 years and 6 years, | 20 | Ages not given, | 2 |
| " 6 " " 12 " | 14 | | |
| Total, | | | 177 |

### NATIVITY.

| | | | |
|---|---|---|---|
| American, | 64 | German, | 41 |
| English, | 7 | Swiss, | 1 |
| Scotch, | 1 | Hollander, | 1 |
| Irish, | 15 | Colored, | 1 |
| Canadian, | 1 | Country not given, | 45 |
| Total, | | | 177 |

